# Prospects for Foamy Viral Vector Anti-HIV Gene Therapy

**DOI:** 10.3390/biomedicines4020008

**Published:** 2016-03-29

**Authors:** Arun K. Nalla, Grant D. Trobridge

**Affiliations:** 1 Pharmaceutical Sciences, College of Pharmacy, Washington State University Spokane, Spokane, WA 99202, USA; arun.nalla@wsu.edu; 2 School of Molecular Biosciences, Washington State University, Pullman, WA 99164, USA

**Keywords:** gene therapy, retroviral vector, foamy viruses, anti-HIV transgenes

## Abstract

Stem cell gene therapy approaches for Human Immunodeficiency Virus (HIV) infection have been explored in clinical trials and several anti-HIV genes delivered by retroviral vectors were shown to block HIV replication. However, gammaretroviral and lentiviral based retroviral vectors have limitations for delivery of anti-HIV genes into hematopoietic stem cells (HSC). Foamy virus vectors have several advantages including efficient delivery of transgenes into HSC in large animal models, and a potentially safer integration profile. This review focuses on novel anti-HIV transgenes and the potential of foamy virus vectors for HSC gene therapy of HIV.

## 1. Introduction

The latest statistics show that nearly 36.9 million people are living with Human Immunodeficiency Virus (HIV) and should be receiving treatment against HIV infection [1]. Highly active antiretroviral therapy (HAART) is the current standard of care which is a combination of drugs that effectively suppress HIV infection and increase the life expectancy of patients [2,3]. A strict adherence to drugs is required to suppress HIV infection and any interruption to a HAART regimen leads to re-emergence of the viral infection. Lifelong adherence to the drugs is also expensive. The annual cost of HAART drugs alone was estimated to be around $10,000–$12,000/patient in the United States, which might even be higher with more aggressive treatment options in patients with treatment failures, due to the development of resistant HIV variants. Importantly, continual usage of these drugs increases the risk of developing metabolic and cardiovascular disorders and non-HIV infection related morbidity [4,5].

The limitations of HAART have led to attempts to provide a permanent one time gene therapy approach using hematopoietic stem cells (HSCs) or T cells as the cell target. The “Berlin Patient” has provided a proof of concept for eradicating HIV infection by transplanting HSCs from an allogeneic donor who is naturally resistant to HIV [6]. This resistance is due to a mutation in the CCR5 allele, a HIV entry coreceptor that occurs in a small minority of the population [7,8,9]. The HSC transplantation approach used to treat the Berlin patient resulted in an effective cure without the need for antiretroviral drugs [7,10]. The Berlin patient has demonstrated the promise of HSC transplantation, but there are major limitations to this approach. The natural occurrence of CCR5 mutation is rare, with only around 1% of the Caucasian population estimated to harbor this mutation in homozygous form [8,11]. Thus, identifying an allogeneic matching donor with a homozygous CCR5 mutation is very rare [11,12], which is supported by the fact that only three additional transplants have been performed since the Berlin patient, but to date only the Berlin patient has had a functional cure against HIV [13]. Another limitation to the Berlin patient approach is that allogeneic transplantation comes with the risk of graft versus host disease. Alternatively, autologous HSC could be genetically modified *ex vivo* to express anti-HIV transgene(s) and infused into patients. HSCs have the ability to self-renew and differentiate into all hematopoietic lineages enabling long-term persistence of the therapeutic anti-HIV transgene(s) in the target cells for HIV infection, primarily CD4^+^ T cells and macrophages. Unlike life-long adherence to HAART, anti-HIV gene therapy could provide permanent protection against HIV with a single treatment. The promise of eradicating HIV by gene therapy is thus of great interest, and efforts to improve the efficacy and safety of HIV gene therapy are being intensively studied.

HSC gene therapy has been used to successfully treat monogenic diseases including adenosine deaminase deficient-severe combined immunodeficiency (ADA-SCID), X-linked SCID, X-linked adrenoleukodystrophy and hemophilia B [14]. In these HSC gene therapy clinical studies retroviral-based vector systems, gammaretroviral (GRV) or lentiviral (LV) vectors, were used to mediate permanent delivery of the therapeutic transgene [15]. Retroviral vectors have been used for HSC gene therapy due to their ability to stably integrate into the host genome, thereby resulting in stable genetic modification of all daughter cells by cell division [16]. This is critically important for HSC gene therapy where there is massive expansion and cell division during hematopoietic differentiation to all blood cell lineages. In the HSC gene therapy setting non-integrating vectors have not yet been effective in large animal models or in patients [15].

Unfortunately, this advantage of retroviral vectors also creates a problem. In the otherwise successful SCID-X1 gene therapy clinical trials, five out of twenty treated patients developed leukemia after treatment. The leukemia was caused due to the GRV provirus integration near proto-oncogenes including LIM Domain Only 2 (Rhombotin-Like 1). This adverse event caused by the integrated vector provirus is commonly referred to as vector mediated genotoxicity. This genotoxicity has raised serious safety concerns for using GRV vectors in HSC gene therapy. However, the clinical benefits achieved for the SCID-X1 trials and other gene therapy trials are compelling, and retroviral HSC gene therapy has moved forward for several diseases [17,18]. There are some challenges that need to be addressed before HSC gene therapy becomes a routine procedure for a wider range of diseases. Safer retroviral vectors need to be developed and thoroughly tested in animal models. To minimize vector mediated genotoxicity, self-inactivating (SIN)-LV vectors have gained favor [19]. Insulators and means to target integration away from proto-oncogenes are also being actively studied to further improve safety. Foamy viral (FV) vectors are particularly promising from a safety standpoint as they have a potentially safer integration profile [20] and are less likely to activate nearby genes than LV or GRV vectors [21].

HIV gene therapy will likely require long-term expression of potent anti-HIV transgenes in target cells. Several anti-HIV transgenes targeting various stages of the HIV life cycle have been identified and evaluated in tissue culture and in animal models. Some have progressed to clinical trials. Expressing multiple anti-HIV transgenes in a combinatorial approach has been more effective to suppress HIV replication than single transgene approaches [22,23,24]. This approach is analogous to the use of drug cocktails in HAART. However the efficient delivery of anti-HIV transgenes in clinical trials has been a major roadblock in part due to the fact that HIV-based LV vector titers are reduced by anti-HIV transgenes [25,26,27]. In this regard FV vectors are also advantageous as they do not share significant homology to HIV, and are not efficiently targeted by anti-HIV transgenes. This review focuses on the potency of anti-HIV transgenes and the potential of FV vectors for delivering anti-HIV transgenes.

## 2. Anti-HIV Transgenes

Anti-HIV transgenes can be either RNA or protein and can interfere either directly with viral components or indirectly with host cellular factors required for viral replication. The anti-HIV transgenes have been grouped into three classes based on where they target the viral life cycle, and mathematical modeling shows each class has very different effects on the HIV life cycle [28]. Class I transgenes inhibit the early stages of the HIV life cycle including viral entry, reverse transcription and integration of the provirus. Class II genes target the post-integration stages and inhibit viral genes that are critical for the translation and production of infectious HIV progeny. Class III transgenes target the late stages of the HIV life cycle, *i.e.*, virion assembly, budding and release. Experimental evidence supports the modeling data that transgenes that block HIV entry are more potent inhibitors than the ones that target post-entry or late stages of HIV replication [28,29]. In the Berlin patient the success of providing a functional cure for HIV was attributed to the lack of a functional CCR5 coreceptor in allogeneic donor cells. Thus the CCR5 mutation which inhibits entry, acts like a class I anti-HIV gene [30]. A gene therapy approach of inhibiting CCR5 expression does have the limitation that it would only block R5-tropic HIV strains that specially use the CCR5 as an entry coreceptor, but not strains that use alternative coreceptors including CXCR4 (X4-tropic). Therefore individual and combinatorial anti-HIV transgenes that target multiple steps in HIV replication and can combat diverse strains of HIV are of great interest. To date, the efficacy of several anti-HIV transgenes have been tested under *in vitro* and *in vivo* settings (Table 1).

### 2.1. Targeting Viral Genes to Inhibit HIV Infection

**Figure 1:**
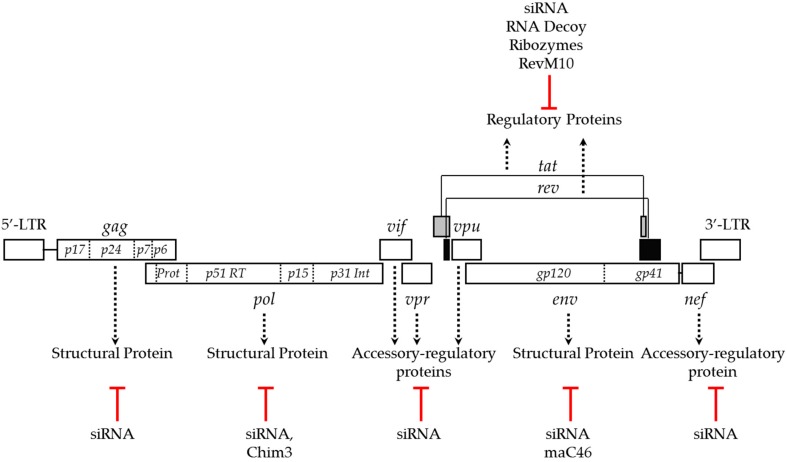
HIV genome and gene therapy approaches targeting viral genes to block replication. Dotted arrows indicate the function of the viral genes and red lines indicate the gene-targeting approaches used to block HIV replication.

The HIV genome encodes multiple genes, including structural, regulatory and accessory genes that play diverse roles at various stages of HIV life cycle. Strategies to suppress the expression or function of these viral genes, including *env*, *gag*, *pol*, *tat*, *rev*, *vpu* and *nef* effectively inhibit HIV infection [29,70,71,72,73] (Figure 1). However, the relative potency of anti-HIV genes varies, and not all anti-HIV transgenes are promising for anti-HIV gene therapy [74,80]. HIV *tat* and *rev* regulate the transcription and translocation of HIV RNA from the nucleus to the cytoplasm, respectively, and have been commonly targeted viral genes for anti-HIV gene therapy. Strategies that include the expression of dominant-negative mutants, ribozymes, RNA decoys and siRNAs targeting *tat* and *rev* successfully inhibited HIV infection in both *in vitro* and *in vivo* models. The trans-dominant negative mutant, RevM10 competes with wild type *rev* for binding to the Rev responsive element (RRE) and inhibits cytoplasmic transport of the HIV transcript. RevM10 was shown to successfully block HIV replication when expressed in T cells and HSCs [61,62]. Ribozymes, small catalytic RNA molecules engineered to cleave specific RNA sequences, targeting *tat* and *rev* were shown to suppress viral transcript levels and inhibit HIV replication in CD34^+^-derived primary monocytes by 1000-fold [63]. Another successful strategy to block *tat* and *rev* activity is the use of small non-coding RNAs as decoys. The HIV Rev protein binds to the RRE of the viral transcript and promotes the translocation of viral RNA across the nuclear membrane into the cytoplasm. Tat, the trans-activator of transcription, is a regulatory protein that efficiently enhances production of viral transcripts by binding to transcription responsive elements (TAR) at the 5′ end of HIV RNA. RNA decoys that mimic RRE and TAR elements significantly interfered with these regulatory proteins and potently inhibited HIV replication up to five-fold [64,65,66,67]. RNAi mediated silencing of the *tat* and *rev* genes efficiently inhibited the expression of the respective proteins, and potently reduced HIV replication in gene modified human T cell lines, primary lymphocytes and CD4^+^ T cells by nearly 1000-fold as determined by p24 levels [68,70]. The success of targeting of *tat* and *rev* in blocking HIV replication allowed this approach to move forward to clinical studies [81,82]. In addition to *tat* and *rev* genes, structural and accessory genes were also targeted to block HIV replication. An approach utilizing siRNA-mediated suppression of viral structural genes, *gag* and *pol* reduced HIV replication by 4–5 logs in SupT1 and ~2–7-fold in PBMC, respectively [75]. Targeting accessory genes such as *nef* also conferred protection against HIV. However, the resistance did not last long due to the emergence of an escape mutant [77]. Recently, published work using CRISPR/Cas mediated targeting of various non-conserved HIV sequences also led to the emergence of escape mutants [69]. Both siRNA- and CRISPR/Cas mediated approaches to inhibit HIV replication confirmed that targeting conserved protein coding viral gene sequences could not only provide long lasting resistance against HIV infection but also delayed the emergence of escape mutants [75,76].

Viral infectivity factor (*vif*) is an auxiliary viral gene that supports HIV infectivity by countering the antiviral activity of the host restriction factor, apolipoprotein B mRNA-editing enzyme catalytic polypeptide (APOBEC)-3G [83]. *Vif* is also a part of HIV reverse transcription complex machinery and promotes reverse transcription and infectivity of HIV [84]. Approaches to disable the *vif* gene have been explored to block HIV replication. Expression of a naturally-occurring *vif* mutant, F12-Vif in CD4^+^ T cells was shown to inhibit HIV infection [85]. Subsequently, a more potent derivative of F-12 Vif, Chim3 was developed. Chim3 acts as dominant negative factor and inhibited the replication of R5- and X4-tropic HIV strains up to six-fold in macrophages and up to 25-fold in CD4^+^ T cells [78,79]. While all of the above anti-HIV transgenes inhibited post entry or late stages of the HIV life cycle, the membrane anchored fusion inhibitor, maC46 is a class I anti-HIV transgene derived from the HIV *env* gene that blocks HIV entry [59]. The maC46 is derived from the C-terminal heptad repeat of HIV gp41 fused to a membrane receptor to express as a membrane-anchored peptide. The maC46 is a 46 amino acid long membrane-bound version of the first clinical approved protein-fusion inhibitor, a 36 amino acid peptide, T20 (Enfuvirtide). T20 potently inhibits HIV replication of various strains, including multiple drug resistant mutants. However, prolonged treatment with T20 results in the emergence of HIV escape mutants that are resistant to T20 based treatment [86,87]. Several derivatives of T20 were subsequently generated that could suppress the replication of T20-resistant mutants [88,89,90]. Like the parental form, maC46 also confers resistance against a broad range of HIV strains [91] when expressed in human cell lines and CD4^+^ T cells [23,27,29,60]. Expression of maC46 was shown to block simian HIV infection up to 4 logs [23]. A direct comparison showed that cells expressing maC46 are highly resistant to HIV infection than cells expressing either anti-*tat/rev* siRNA or antisense RNA against *env* [29]. Though potent, emergence of escape mutants against T20 raises concerns for using maC46 based treatment. Strategies targeting viral genes, especially monotherapies targeting a single gene, are prone to the emergence of escape mutants due to the low fidelity of HIV reverse transcriptase. In order to prevent viral escape, strategies targeting either a host gene that facilitates HIV replication or that target multiple genes have been employed.

### 2.2. Using Host Cellular Proteins as Targets to Block HIV Infection

**Figure 2:**
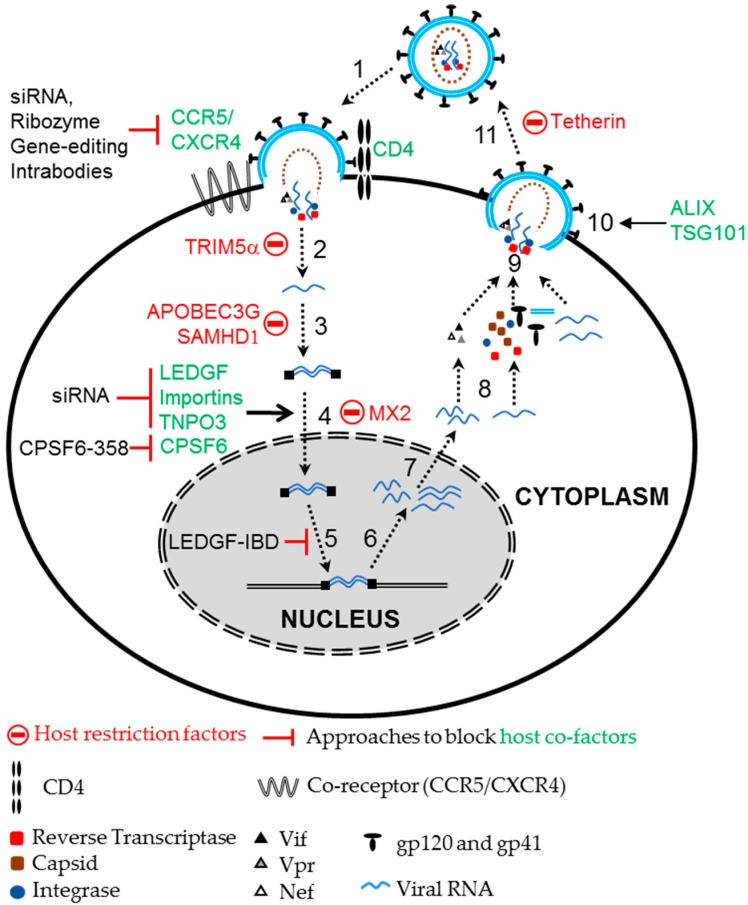
HIV-1 life cycle and host co-factors. HIV exploits several host co-factors for its infection and replication. For entry, HIV uses the host receptor CD4 with the coreceptors CCR5 or CXCR4. Nuclear import and integration of the viral genome is supported by several host co-factors including LEDGF, Importin and TNPO3. Host cell cycle regulators support viral transcription, while proteins like ALIX and Tsg101 aid in budding of HIV virions. In contrast, HIV infection is restricted by several host restriction factors like TRIM5α, SAMHD1, APOBEC3G, Tetherin and MX2. Altering these co-factors and restriction factors by various strategies like siRNA, gene editing, ribozymes, expression of dominant negative variants and host restriction factors can block HIV replication. Numbers represent the steps in HIV life cycle, 1: Entry; 2: Uncoating; 3: Reverse Transcription; 4: Nuclear import; 5: Integration; 6: Transcription; 7: Nuclear export; 8: Translation; 9: Assembly; 10: Budding; and 11: Release and maturation.

HIV depends on host factors to facilitate entry and integration of the viral genome, as well as transcription and translation of viral proteins in target cells (Figure 2). Disrupting the function or expression of these host genes can also block HIV infection. Targeting viral entry provides an effective way to interfere with HIV infection and CD4 is the primary receptor used by HIV to invade target cells. HIV uses additional coreceptors, primarily CCR5 or CXCR4 that determine the tropism, R5- and X4-tropic HIV strains for T cells and macrophages, respectively. Therapeutic approaches targeting these receptors have been developed to block HIV infection. Targeting CD4 is not as attractive as targeting CCR5 because of its essential role in immunological functions. However, strategies to block the interaction between the viral gp120 envelope protein and cellular CD4 receptor by expressing soluble CD4 [92], anti-CD4 monoclonal antibodies [93] and CD4-based bi-specific chimeric antigen receptors [94] all inhibited HIV entry. Strategies targeting HIV entry coreceptors have been extensively studied, with CCR5 being the most commonly targeted coreceptor [31,32,33]. Several strategies to disrupt CCR5 expression including intrabodies, ribozymes, siRNAs and gene editing approaches using zinc finger nucleases (ZFNs) and CRISPR/Cas have been evaluated. Blocking CCR5 expression by retaining CCR5 receptors in the endoplasmic reticulum with CCR5-specific single chain intrabodies protected lymphocyte cell lines, CD4^+^ T cells, and thymocytes against HIV infection *in vitro* [34]. Ribozymes targeting CCR5 efficiently suppressed CCR5 expression and inhibited HIV replication [35,36]. siRNA and miRNA based targeting of CCR5 mRNA effectively blocked HIV infection *in vitro* and *ex vivo* in xenotransplant mouse models [37,38,39]. Permanent disruption of the CCR5 allele using gene editing tools like ZFNs in T cells [40] and CD34^+^ cells [41] conferred resistance against R5-tropic HIV infection. A gene editing approach using the most recently discovered CRISPR/Cas system was also used to permanently disrupt the CCR5 allele [42,43]. Following allelic disruption of CCR5 via CRISPR/Cas and a CCR5 specific small guide RNA in induced-pluripotent stem cells, the cells were allowed to differentiate into monocytes and macrophages. The differentiated CCR5 mutant cells were resistant to HIV infection [42]. Wang and coworkers showed that efficient disruption of CCR5 by CRISPR/Cas in human CD4^+^ T cells confers resistance against R5-tropic HIV infection [44]. Some of these approaches targeting CCR5 have been evaluated in clinical trials [95,96]. Targeting CXCR4, an entry coreceptor used by X4-tropic strains, by siRNA- and CRISPR/Cas-mediated approaches was shown to confer resistance against X4-tropic HIV infection [45,46,47]. Unlike CCR5, CXCR4 is widely expressed in a variety of cells and is known to play a significant role in several physiological processes including stem cell homing [97,98]. CXCR4-null mice display severe hematopoietic and nervous disorders including reduced B-cell lymphopoiesis, myelopoiesis, and cerebellum development which often results in death at perinatal stage [99]. Conversely, CCR5-negative humans seems to have no deleterious effects [11]. Thus, the approach of targeting CCR5 is more attractive than targeting CXCR4.

Following entry, the virion uncoats, allowing the viral RNA to be reverse transcribed, imported into the nucleus to integrate into the host genome and continue its life cycle. HIV depends on various host factors for nuclear import and integration in the host cell genome. Lens epithelium derived growth factor (LEDGF) is a co-factor of HIV integrase that aids nuclear import of the viral pre-integration complex and supports chromosomal tethering of HIV [100,101,102]. Depleting LEDGF or blocking the interaction between LEDGF and the HIV integrase, either by expressing the integrase binding domain (IBD) of LEDGF or a small molecular inhibitor, LEDGIN, reduced chromosomal integration of HIV [50,51,103]. Expression of the IBD was shown to compete with endogenous LEDGF, interfere with HIV integration, and block HIV replication by more than 100-fold [103]. Stable expression of both the LEDGF-IBD, a peptide that lacks the chromatin binding domain, and a siRNA targeting LEDGF inhibited HIV infection in human cell lines and CD4^+^ T cells by 40-fold [52]. Mice engrafted with human CD4^+^ T cells expressing LEDGF/IBD showed significantly reduced levels of HIV load compared to mice engrafted with control CD4^+^ T cells upon HIV challenge [52]. HIV also depends on various other host factors such as importins and transportin 3 (TNPO3) for nuclear import [104,105]. Knockdown of importin-7 inhibited nuclear import of the HIV genome in macrophages [106], but had no effect on HIV replication [107]. A study by Zielske and Stevenson demonstrated that Importin-7 plays a critical role in accelerating the nuclear import of HIV [107]. Similarly, TNPO3 was also shown to be a key component of nuclear entry by various reports [108], although this function is debated [109]. Depletion of TNPO3 blocks nuclear entry and inhibits HIV replication, and this is dependent on another host protein, cleavage and polyadenylation specificity factor 6 (CPSF6) [109,110]. CPSF6, a nuclear protein, interacts with the HIV capsid protein and is implicated in regulating nuclear entry of HIV. However, suppression of endogenous CPSF6 did not affect HIV replication *in vitro*. The expression of a C-terminal truncated version of CPSF6 (CPSF6-358) was enriched in the cytoplasm, targeted the viral capsid protein and restricted nuclear import of HIV, inhibiting replication [55,56]. The anti-HIV efficacy of CPSF6-358 was shown to be equivalent to that of TRIM5α. Expression of CPSF6 blocked the replication of various HIV strains, but did not block the replication of variants with a N74D mutation in the HIV capsid protein [55].

Other cellular factors like autophagy related 16-like (Atg-16), autophagy related 5-like (Atg-5), Heat Shock 60 kDa protein (Chaperonin), TSG101, and ALIX which supports various stages of the HIV life cycle, were also shown to block HIV replication. A study to determine the effects of 30 known cellular factors on HIV replication demonstrated that cell lines expressing siRNA against ALIX, Atg-16 and TRBP genes were resistant to HIV replication for up to two months [53]. A subsequent study demonstrated that knockdown of various autophagy factors including Beclin-1, WIPI-1, PIK3R4, Atg-4, Atg-5, and Atg-16, inhibited HIV replication by blocking the production of viral particles. Further, the authors showed that simultaneous knockdown of two autophagy related proteins, Atg-5 and Atg-16 could delay HIV replication in SupT1 cells up to 100-fold compared to control cells [54].

In addition to host factors that support HIV replication, several host factors are known to restrict viral replication [111]. These include tripartite motif-containing protein 5 alpha (TRIM5α), APOBEC3G, tetherin and SAMHD1 (SAM domain and HD domain-containing protein 1) [112,113,114,115,116,117,118]. Strategies targeting these host restriction factors to block HIV replication have also been explored. In turn HIV expresses several accessory proteins to counter the antiviral activities of host restriction factors [111]. The viral accessory genes, *vif*, *vpx* and *vpu* were shown to counter the anti-viral restriction activities of APOBEC3G, SAMHD1 and Tetherin, respectively. Nonhuman primate TRIM5α confers potent resistance against HIV infection in old world monkeys [112]. Although the inhibitor mechanism is not fully understood, nonhuman primate TRIM5α binds to the viral capsid and blocks uncoating of HIV virions in old world monkeys. Human TRIM5α only modestly restricts HIV replication but ectopic expression of *Rhesus macaque* TRIM5α in human cells efficiently restricted HIV infection [112]. Similarly, a chimeric TRIM5α-CyclophilinA (AoT5Cyp) fusion found in the *Aeotus* genus of the new world owl monkey inhibited HIV infection when expressed in human cells [119]. Expressing a non-human transgene in humans has the potential to generate an immune response to the foreign transgene, which has hindered the use of nonhuman primate TRIM5α as an anti-HIV transgene. However, altering a small number of amino acids in the active or the capsid binding domains of human TRIM5α resulted in effective restriction of HIV infection in human cells [48,120,121,122,123]. Neagu and coworkers engineered a humanized version of TRIM5α-CyclophilinA fusion (hT5Cyp) that was able to restrict HIV infection in human cells at a comparable efficiency to the potent Rhesus/human chimeric AoT5CypA [49]. When hT5CypA is expressed in primary CD4^+^ T cells and macrophages it confers a survival advantage upon HIV infection *in vitro*. Transplantation of hT5CypA expressing CD4^+^ T in a mouse xenotransplant model resulted in effective engraftment and protection against HIV infection. Subsequently, another fusion protein of TRIM21 and CyclophilinA was also shown to possess potent anti-HIV inhibition similar to hT5CypA [124]. It remains to be determined if these proteins will be immunogenic in the setting of HSC transplantation in patients. Given the fact that allogeneic cells can be used for HSC transplantation, it is likely that immune responses to specific transgenes could be managed.

Host factors stimulated by type-1 interferon (IFN) upon viral infection also inhibit HIV replication [125,126]. The IFN stimulated gene, human myxovirus resistance 2 (*MX2*) has been shown to inhibit HIV replication [57,58]. MX2 was demonstrated to be a late post-entry suppressor of HIV, which blocks nuclear import of sub-viral complexes. Ectopic expression of MX2 was shown to potently inhibit the replication of several laboratory HIV isolates [57]. In summary, there are many anti-HIV transgenes available for an HSC gene therapy approach. Designing safe and effective vectors that express multiple transgenes in a combinatorial gene therapy approach is an important goal to bring HIV gene therapy to the clinic.

### 2.3. Efficacy of Combinatorial Approaches to Combat HIV Infection

Anti-HIV gene therapy is expected to be more effective when a combinatorial approach targeting multiple stages of the HIV life cycle is used. This increases the potency due to expression of multiple anti-HIV transgenes in the same cell and also can prevent the emergence of escape mutants. Kimpel and colleagues evaluated the potency of three anti-HIV transgenes, an anti-*tat*/*rev* siRNA, RNA antisense against the viral envelope, and maC46. They found that cells expressing maC46 and *env* RNA antisense strongly inhibited HIV infection in *in vitro* conditions [29]. Cells expressing maC46 had a better survival advantage over the cells expressing *env* RNA antisense and anti-*tat/rev* siRNA in a humanized xenotransplant mouse model upon HIV infection. Although the anti-*tat*/*rev* siRNA was less potent, their combination with entry inhibitors such as anti-CCR5 and maC46 expression improved the overall potency of the combinatorial construct [23,127]. Further, the protection developed by single anti-HIV transgene is more likely to fail as the result of the development of an escape mutant or phenotypic switch [77,128]. Escape occurs due to the error prone reverse transcriptase of HIV. Phenotypic switching between R5- to X4-tropic HIV strain during late stages of infection, resulting in emergence of X4 tropic strains in the patients treated with anti-CCR5 drugs occurs frequently [129,130,131]. Targeting both CCR5 and CXCR4 could provide a broader protection against diverse strains but targeting CXCR4 is problematic, as described above. Simultaneous expression of multiple anti-HIV transgenes targeting various HIV genes showed broad resistance against diverse HIV isolates and can delay the emergence of escape mutants [75,128].

For HSC gene therapy a combinatorial anti-HIV transgene cassette must be efficiently and stably delivered to a HSC. To ensure each transduced HSC gets all the transgenes, a single vector with multiple transgenes has commonly been used. Several groups have designed a single vector comprising a gene cassette that express multiple anti-HIV genes targeting various stage of the HIV life cycle. Including at least one class I anti-HIV transgene that targets viral entry has proved to be highly effective [22,27,75,76]. A combinatorial vector expressing a TAR decoy and an anti-CCR5 ribozyme displayed improved resistance against HIV-infection relative to expressing a TAR decoy alone both *in vitro* and *in vivo*. Further, a preclinical study revealed that the transgenes from the above combinatorial vector did not interfere with thymopoiesis [22]. The addition of a third anti-HIV transgene, *i.e.*, *tat/rev* siRNA, to the above combinatorial anti-HIV transgenes (anti-CCR5 ribozyme and TAR decoy) had better anti-HIV effect than each anti-HIV transgene alone [127]. Cells transduced with the triple combinatorial anti-HIV vector suppressed HIV replication by nearly 2 logs [127]. Subsequent studies showed that human CD34^+^ cells transduced with the same triple combinatorial anti-HIV transgenes were able to differentiate into T cells in mice. The differentiated transgene-expressing T cells were resistant to HIV infection by more than 10-fold when challenged *ex vivo* [132]. In yet another approach, CD34^+^ cells transduced with a vector expressing three highly potent anti-HIV transgenes, *i.e.*, CCR5 siRNA, chimeric TRIM5α and TAR decoy were shown to efficiently inhibit HIV replication [133]. Importantly, the expression of the above combinatorial anti-HIV transgenes significantly delayed the emergence of escape mutants as evaluated by long-term culture of challenged cells. The emergence of escape mutants can also be avoided by using a combinatorial approach of targeting conserved protein coding viral sequences. Simultaneous targeting of viral genes including a combination of *gag*, *pol*, *nef* and *tat/rev* by siRNA-mediated suppression provided long lasting resistance against HIV infection and also delayed the emergence of escape mutants compared to targeting a single viral gene [75,76,134].

In yet another combinatorial approach, CD34^+^ derived macrophages transduced with a combinatorial vector expressing the maC46 and anti-*tat/rev* siRNA were highly resistant to HIV infection and had a survival advantage upon HIV challenge compared to cells transduced with vector expressing individual anti-HIV transgene. Cells transduced with a triple combinatorial vector expressing anti-*tat/rev* siRNA, anti-CCR5 siRNA and maC46 showed significantly better protection against HIV infection than cells expressing the maC46 alone [23]. A preclinical study demonstrated that human CD34^+^ cells transduced with a dual combination anti-HIV vector, expressing CCR5 siRNA and maC46, could efficiently support long term engraftment and differentiation into multilineage hematopoietic cells in a humanized mouse model and provide resistance against both R5- and X4-tropic HIV infection [135,136].

## 3. Anti-HIV Gene Therapy Clinical Trails

The success of gene therapy approaches in preclinical models led to clinical trials to test the efficacy of anti-HIV gene therapy approach in patients. These clinical trials evaluated therapeutic benefits and also examined the safety, transduction efficacy, engraftment, immunological tolerance and long-term persistence of the gene modified cells in the infused patients. Moloney murine leukemia viral based GRV or LV vectors have been used to deliver the anti-HIV transgene by *ex vivo* transduction of either human CD34^+^ cells or T cells. The anti-HIV transgenes evaluated in clinical trials include: RRE decoy [137], RevM10 [81,138], anti-*tat/vpr* ribozyme [82,139], maC46 [140], CCR5 gene editing by ZFN [96] and a triple combinatorial anti-HIV gene cassette expressing anti-CCR5 ribozyme, anti-*tat/rev* siRNA and TAR decoy [95].

The efficacy of RRE decoy was tested in pediatric HIV patients. Patients infused with GRV vector-mediated modified autologous bone marrow derived CD34^+^ cells expressing a RRE decoy showed no evidence of adverse effects on patients. However, gene marking in peripheral blood samples was negligible after one year of transplantation [137]. This study highlighted the importance of improving gene transfer and engraftment efficiency [137]. Similarly, an anti-*tat* ribozyme (RRz2) anti-HIV HSC gene therapy was evaluated in phase I clinical studies [82,139]. Results showed that the gene modified T cells expressing RRz2 were not eliminated from the patient and expression of RRz2 was detected in the gene modified cells up to 4 years, but at a very low frequency [82]. Gene marking in peripheral blood samples in the patients ranged from 0.1% to 0.005% at 24 week point after infusion. A phase II study with seventy-four HIV patients evaluated a class II OZ1 (*tat*/*vpr* ribozyme, referred previously as RRz2) transgene delivered by a replication-incompetent GRV vector into CD34^+^ cells. Similar to the phase I study, transplantation of OZ1 transduced HSC showed no adverse clinical implications. Though gene marking and OZ1 expression was detectable in peripheral blood cells, the levels never reached the quantifiable range. Moreover, OZ1 modified cells that were detectable in 94% of the participants after four weeks of infusion gradually decreased to 7% at 100 weeks. In spite of this low gene marking and expression, the CD4^+^ T cell counts were higher in OZ1 treatment patients than the placebo group throughout the 100 weeks [141]. However, these trials were limited by very low marking.

The efficacy of Class I anti-HIV transgenes have also been evaluated in human trials. A German clinical trial employed infusion of T cells transduced with a GRV vector expressing the potent maC46 anti-HIV transgene to treat ten HIV-patients with advanced disease [140]. Infusion of T cells expressing maC46 did not result in any detectable immune response to the foreign transgene but had low gene marking, below 0.01% in total leukocytes after seven days. Gene modified cells were detectable in peripheral blood, lymph node and bone marrow up to one year post infusion but gradually decreased over time and was very low at one year (ranged below 0.001%–0.0001%). However, due to a lack of any significant change in HIV load during the first 4 months post infusion, seven out of the ten patients returned to a HAART regimen. Interestingly, four out of seven patients showed low viral loads, however the reason for this outcome is not clear.

The safety and the tolerability of a conditionally replicating LV vector expressing an antisense gene against the HIV envelope has also been evaluated in a phase I clinical trial [142]. A conditionally replicating LV vector retained the complete 5′- and 3′-LTR and all the cis-regulatory elements required for retroviral replication that replicated only in presence of wild type HIV, *i.e.*, by HIV infection in the transduced cells. Five patients with chronic HIV infection were infused with gene-modified autologous CD4^+^ T cells. Cells expressing the antisense *env* were well tolerated in all patients. The engraftment frequency ranged from 0.023% to 0.04% after two years of infusion. CD4^+^ counts in four out the five patients either remained stable or increased; and at least one patient showed sustained decrease in the viral load. In yet another clinical study, the safety of a combinatorial anti-HIV gene therapy approach was evaluated, in this case a SIN-LV vector was used [95]. Four AIDS/lymphoma patients were transplanted with HSCs transduced with SIN-LV vector with combinatorial anti-HIV gene cassette expressing: anti-CCR5 ribozyme, anti-*tat/rev* siRNA and a TAR decoy. Expression of the anti-HIV transgenes were detectable for up to two years, but the gene marking frequency in the peripheral blood mononuclear cells was low and ranged between from 0.02%–0.32%. A more recent anti-HIV gene therapy trial evaluated the efficacy of infusing autologous CD4^+^ T cells that were gene-edited to disrupt CCR5 by ZFNs [96]. CCR5 disrupted CD4^+^ T cells readily engrafted and persisted long-term without any adverse events. This study showed a survival advantage of CCR5 disrupted CD4^+^ T cells in the HIV infected patients.

These clinical trials have demonstrated that an HSC based anti-HIV gene transfer approach to combat HIV infection is potentially safe and feasible, but the very low gene marking observed highlights a need for more effective ways to deliver transgenes with improved engraftment levels [143]. Therefore, efforts to develop improved vectors and transplantation protocols are needed to advance HSC anti-HIV gene therapy [143].

## 4. Retroviral Vectors and Their Limitations in Anti-HIV Gene Therapy

The ability to efficiently transfer and stably express the therapeutic transgene in the target cell is critical for HSC gene therapy. To date, retroviral vectors (GRV and LV) have been the vectors of choice to deliver therapeutic transgenes to HSCs. Retroviral vector-mediated gene therapy has been used successfully to treat hematologic diseases including SCID-X1, X-linked chronic granulomatous disease (CGD), Wiskott-Aldrich syndrome and β-thalassemia, and was also used in an anti-HIV gene therapy clinical study [95,144].

### 4.1. Gammaretroviral Vectors

Most early HSC gene therapy studies used GRV vectors including HIV clinical trials. However, HIV gene therapy clinical studies were limited by low gene marking. This was in part can be attributed to the low efficiency with which GRV vectors transduce non-dividing cells [145]. GRV vectors require mitosis for transduction, making quiescent HSCs refractory to transduction with GRV vectors. Additionally, the adverse side effects from the SCID-X1 gene therapy trials have highlighted safety problems with GRV vectors. Following the adverse events, studies were conducted to show GRV vectors integrate close to the transcription start sites of genes which can lead to activation of proto-oncogenes [17,18,146,147]. This has led to a search for alternative retroviral vectors that can efficiently transduce HSCs and have a safer integration profile.

### 4.2. Lentiviral Vectors

LV vectors, based on HIV retroviruses, transduce non-dividing cells more efficiently than GRV vectors [148,149,150]. Gene therapy studies using LV vectors have showed promise in treating diseases such as X-linked adrenoleukodystrophy and β-thalassemia. High throughput analysis of LV integration sites in the gene modified cells from patients treated for X-linked adrenoleukodystrophy and HIV gene therapy studies indicated neither clonal dominance [151] nor an enrichment of integrations in or near proto-oncogenes [142]. However, the patient treated for β-thalassemia showed an expansion of a clones with provirus integrations in the *HMGA2* locus. These integrations caused transactivation and read-through transcription of the *HMGA2* gene, resulting in increased levels of HMGA2 in a dominant clone [152]. Though the patient remained healthy with no signs of serious adverse effects, as of now, the ability to integrate within genes and transactive these genes is a significant safety concern.

### 4.3. Limitations of HIV Based LV Vectors for Anti-HIV Gene Therapy

The potential of LV vectors for anti-HIV gene therapy has another limitation: the phylogenetic relation of the LV vector to HIV. LV vectors are HIV based and this evolutionary link not only raises safety concerns related to heterologous recombination between the LV vector and wild type HIV, but can also affect LV vector production. Although LV vectors were shown to efficiently carry various anti-HIV transgenes and block HIV replication, certain anti-HIV genes that share sequence or functional similarity with LV and the use of multiple anti-HIV transgenes can severely reduce titers of the anti-HIV LV vectors [25,26,74,75,80]. Anti-HIV transgenes that target HIV *tat/rev* significantly reduce LV titers up to 200-fold and a combinatorial anti-HIV cassette expressing *tat/rev* siRNAs, maC46 and RevM10 reduced the titers up to 1500-fold [27]. The expression of siRNAs targeting *gag* and *pol* sequences negatively affected LV vector production by dropping the titers up to 10-fold [74]. However, the use of human codon optimized *gag* and *pol* packaging plasmids improved the titers of anti-HIV LV vector targeting *gag* and *pol*. In addition, several other mechanisms including Drosha cleavage, the presence of constitutive promoters, like cytomegalovirus promoter, and poly(A) signals in the vector system were also reasoned to reduce the titers of anti-HIV miRNA containing LV vectors [80]. Clearly, LV vectors will have limitations in terms of their ability to efficiently deliver these transgenes. Another concern is the possibility of vector mobilization due to the recombination with wild type HIV, which might result in adverse consequences [153,154], however, using SIN-LV vectors has reduced the chances of vector mobilization [26].

Lentiviral vectors that are not based on HIV could avoid the impact of anti-HIV transgene on LV vector production. Vectors derived from simian immunodeficiency viruses (SIV) and nonprimate LVs such as feline immunodeficiency viruses and equine infectious anemia virus have also been developed for HSC gene therapy. However, genetic recombination and cross packaging between phylogenetically distinct retroviruses has been documented which is a concern for using these LV in anti-HIV gene therapy [155,156,157]. Moreover, a study by Browning and coworkers demonstrated that the LV belonging to different subfamilies are functionally more similar than originally thought, and upon coinfection cross-packaging between related retroviruses can generate distinct viruses or chimeric variants with unknown pathogenic potential [158].

## 5. Foamy Viral Vectors

FV vectors have shown a great promise for HSC gene therapy [159]. FV are member of *Spumaretrovirinea*, a subfamily of retroviruses. FV are endemic in various non-human primates, cats, cattle and horses, and have co-evolved with their natural hosts. Although, humans are not a natural host, FV has been detected in humans through zoonotic infection. FV infection has not resulted in any disease in either natural or accidental hosts, including humans [160]. Moreover, FV replication is unique in that reverse transcription occurs during the late stage of FV life cycle resulting in packaging of DNA in infectious virions, rather than RNA. However, the relative amount of RNA and DNA in FV virions is still debated. FV have a relatively large genome allowing FV vectors to efficiently deliver transgenes up to 9.2 kb [161]. The wide cell tropism mediated by the FV envelope allows FV vectors to transduce essentially any mammalian cells, including dog HSC and human CD34^+^ cells with high efficiency [162,163,164,165,166]. FV vector transduction requires mitosis but FV vectors can persist as a stable transduction intermediate in quiescent cells and can transduce quiescent G_0_ cells at similar efficiency to LV vectors [167]. FV vectors compare favorably to LV vectors for HSC transduction in the large animal dog model [168] and in human CD34^+^ cells, and capable of engrafting in immunodeficient mice [169]. FV vectors also appear safe and effective when tested in the large animal, dog model. FV vector mediated HSC gene therapy was able to provide a functional cure for pyruvate kinase deficiency [170] and leukocyte adhesion deficiencies in dogs [171,172]. The poor safety profile of GRV vectors and the close phylogenetic relationship between LV vector and HIV has led to the development of FV vectors for anti-HIV HSC gene therapy. FV vectors efficiently transduced HSCs and can efficiently deliver some anti-HIV transgenes that lower the titer of LV vectors.

### 5.1. FV Vectors Have a Promising Safety Profile

Vector-mediated genotoxicity is a major concern of using retroviral vectors in gene therapy approach. LV vectors prefer to integrate within genes [20,173] and can generate chimeric transcripts by read-through transcription [21]. Proviral integration profile analysis and genotoxicity studies suggest FV vectors may be safer than other retroviral vectors. FV vectors integrate near transcription start sites less often than GRVs and integrate within genes less often than LV vectors [20,169,172,173]. A genotoxicity study comparing the ability of retroviral vectors to transactivate nearby genes revealed that FV vectors are less likely to transactivate nearby genes compared to GRV and LV vectors [21]. A high throughput integration site analysis of FV vector transduced long-term repopulating gene-modified cells in dog and mouse models showed no signs of clonal dominance [171,172,174]. Together, these studies support the use of FV vectors for anti-HIV gene therapy.

### 5.2. Anti-HIV Transgenes in FV Vectors

Another important feature that favors FV vectors to HIV-based LV vectors for anti-HIV gene therapy is the lack of homology to HIV. At least two studies revealed that anti-*rev* siRNA expression significantly drops LV viral vector titer up to 2 logs, whereas the same anti-HIV transgene had no significant effect on the titers of FV vectors [23,27]. Therefore, unlike LV vectors, anti-HIV transgenes are less likely to inhibit FV vector production and reduce vector titers. High titer vector is crucial for translation to the clinic.

### 5.3. FV Vectors for Anti-HIV Gene Therapy

The efficacy of various anti-HIV transgenes in a FV vector backbone and in stem cell based anti-HIV gene therapy have been explored [23,27,175]. The ability to block SIV infection, a close relative of HIV, by expressing anti-*tat/rev* siRNA encouraged the use of FV vectors in expressing anti-HIV transgenes and its utility in anti-HIV gene therapy [176]. Park and colleagues designed a FV vector expressing a combinatorial anti-HIV miRNA cassette targeting two regions of the HIV LTR and HIV-*rev* under the control of a Tat-inducible heat shock protein promoter [175]. Expression of anti-HIV miRNAs inhibited HIV replication in an *in vitro* HIV challenge assay by more than 98%. This study demonstrated that combinatorial anti-HIV transgene cassettes expressing TAR and anti-*rev* siRNA or TAR, anti-*rev* and anti-LTR siRNAs had showed an increased ability to block HIV replication than the cell expressing TAR alone [175]. Yet another study revealed the efficacy of FV vectors expressing combinatorial anti-HIV transgenes in inhibiting HIV replication [27]. CD34^+^ derived macrophages transduced with either maC46 or anti-*tat/rev* siRNA or the anti-HIV combinatorial cassette expressing RevM10, anti-*tat/rev* siRNA and maC46 showed ~4 logs reduction in HIV infection relative to untransduced and cells transduced with RevM10 alone. Benefits of the combinatorial anti-HIV transgene were further evaluated using an *in vitro* competitive survival advantage assay. The results revealed that cells expressing triple combinatorial anti-HIV transgenes had ~5.2 times more surviving cells after 16 days of HIV challenge, compared to cells expressing either anti-*tat*/*rev* siRNA or RevM10.

Similarly, Kiem and colleagues revealed that lymphocytes expressing multiple anti-HIV transgenes including maC46, siRNAs against *tat/rev* and CCR5, were highly resistant to HIV infection [23]. In a single-cycle infection assay, the authors observed that cells transduced with FV vector expressing maC46 or anti-HIV combinatorial FV vector expressing maC46, siRNAs against *tat/rev* and CCR5, showed ~20- and ~23-fold reduction in HIV infection compared to cells transduced with a control FV vector. This study was first to use an FV vector with a P140K mutant of methylguanine methyltransferase gene (MGMT^P140K^) in mediating *in vivo* selection of human repopulating cells with an anti-HIV transgene in a mouse model. Expression of MGMT^P140K^ makes the cells resistant to O6-benzylguanine (O6BG), but functionally active to counter the effect of the bis-chloroethyl nitrosourea (BCNU), a chemotherapeutic drug. Administration of BCNU and O6BG selectively kills untransduced cells and allows selective enrichment of transgenic cells expressing MGMT^P140K^. This approach of *in vivo* selection was successful in increasing the percentage of gene marked cells and in expansion of gene modified repopulating cells [23,174]. In summary, the non-pathogenicity to humans, ability to transduce HSC and promising integration profile support FV vectors for anti-HIV gene therapy.

### 5.4. FV in HIV Vaccine Development

In addition to the gene therapy applications, FV vectors have also been explored for vaccine development including HIV vaccines [177]. Viral vaccines are derived from pathogenic viruses which are attenuated or inactivated and carry epitopes to elicit an immune response upon immunization. Replication-competent FV have also been used as vaccines. Though non-pathogenic, FV showed persistent infection in various hosts and induced strong cellular and humoral immune responses. The combination of being non-pathogenic, immunogenic, and the ability to integrate in the host and replicate in lymphoid cells has the potential to result in prolonged epitope expression eliciting a robust host immune response. Replication-competent FV vectors carrying various epitopes were designed to induce specific antibodies against their epitopes [178]. Gag, Env and Bet proteins of FVs were used as epitope carriers. The conserved domains in the above FV genes were replaced with the epitope sequence of the pathogen. Among the three, alterations in Bet protein had the least impact on FV capsid formation, virions release and viral titers [178]. For the successful development of a HIV vaccine, the epitope used should able to induce a broad array of neutralizing antibodies that prevent infection and the integration of the viral genome into target cells. Several neutralizing antibodies against surface and transmembrane (TM) and membrane proximal external region (MPER) of HIV gp41, were isolated from HIV infected patients. A Chimeric FV vector expressing TM and MPER HIV epitopes was designed that could successfully induce antibodies against HIV in a rat model [177]. In this study, the FV Bet protein was altered by replacing conserved regions with MPER of HIV gp41 proteins. The epitope expressed by the chimeric FV vector readily reacted with 2F5 and 4E10 HIV antibodies *in vitro*. Further, rats immunized with the chimeric FV vector induced antibodies specific to HIV gp41 that could bind to HIV particles. However, the antibodies induced by the chimeric FV vector showed no effect on HIV infection when tested in an indicator cell-line based neutralizing assay. While the lack of neutralizing effects by these antibodies requires further testing, this study opened up the feasibility of using a FV vector for HIV vaccines.

## 6. Conclusions and Future Directions

Gene therapy has the potential to offer a life-long therapeutic option for controlling HIV infection. However, there are several challenges that must be overcome before anti-HIV gene therapy can become a widely used therapy to treating HIV patients. First, anti-HIV transgenes that can inhibit diverse HIV strains and inhibit escape are needed. Expressing multiple anti-HIV transgenes, especially targeting viral entry, in the same cells using a single combinatorial anti-HIV vector, is a feasible approach to increase the efficacy of blocking HIV that can even reduce the chance of the emergence of escape mutants. The success of HIV gene therapy, depends on the efficient engraftment and long-term persistence of gene modified cells in a human system. For this, safe and efficient delivering systems are needed for *ex vivo* transduction of HSC. FV vectors have several advantages for HIV HSC gene therapy, including efficient transduction of HSCs, efficient delivery of anti-HIV transgenes and a promising safety profile.

## Figures and Tables

**Table 1:** Anti-HIV transgenes and strategies used to block the expression.

Target Type	Gene	Function Related to HIV Life Cycle	Interacting Stage	Inhibition Strategies	Inhibitory Class	References
Host	*CCR5*	Entry coreceptor	Entry	siRNA, Ribozymes, Gene-editing, Intrabodies	I	[31,32,33,34,35,36,37,38,39,40,41,42,43,44]
	*CXCR4*	Entry coreceptor	Entry	siRNA, Gene-editing	I	[45,46,47]
	*TRIM5*	Host restriction factor	Uncoating	Protein expression	I	[48,49]
	*LEDGF*	Interacts with viral integrase and transport pre-integration complex into nucleus	Nuclear import, Pre-integration	siRNA, Expression of LEDGF-IBD	I	[50,51,52]
	*Atg-5* & *Atg-16*	Autophage related	Entry or early RT	siRNA	I	[53,54]
	*CPSF6*	Host cofactor bind to viral capsid protein	Nuclear import	expression of CPSF6-358	I	[55,56]
	*MX2*	Interferon stimulated protein	Uncoating, nuclear import	Protein expression	I	[57,58]
Viral	*maC46*	Membrane anchored protein-fusion interferes with gp41 and block viral fusion to cell membrane	Entry	Protein expression	I	[23,27,29,59,60]
	*RevM10*	Dominant negative mutant competes with viral *rev*	Post-integration	Protein expression	II	[61,62]
	*rev*	Translocation of viral transcripts from nucleus to cytoplasm	Post-integration	siRNA, Ribozymes, RNA decoy, CRISPR/Cas	II	[61,62,63,64,65,66,67,68,69]
	*tat*	Viral transcription regulatory protein	Post-integration	siRNA, Ribozymes, RNA decoy, CRISPR/Cas	II	[61,62,63,64,65,66,67,68,69]
	*gag-pol*	Viral enzymes: reverse transcriptase, integrase, RNaseH and protease	Reverse transcription and pre-integration and assembly	siRNA, CRISPR/Cas	I	[69,70,71,72,73,74,75,76]
	*nef*	Accessory protein, modulate CD4 expression and stimulate HIV infectivity	Assembly, Budding	siRNA CRISPR/Cas	III	[69,76,77]
	*Chim3*	Derivative of a mutant viral infectivity factor, (F12-Vif)	Pre-integration	Protein expression	I	[78,79]
